# Functional and occupational characteristics predictive of a return to work within 18 months after stroke in Japan: implications for rehabilitation

**DOI:** 10.1007/s00420-013-0883-8

**Published:** 2013-05-16

**Authors:** Hirotaka Tanaka, Toshihiro Toyonaga, Hideki Hashimoto

**Affiliations:** 1 Department of Rehabilitation, Chubu Rosai Hospital, Japan Occupational Health and Welfare Organization, 1-10-6 Komei, Minato-ku, Nagoya, 455-8530 Japan; 2 Clinical Research Center for Worker’s Rehabilitation, Japan Occupational Health and Welfare Organization, Kitakyushu, Japan; 3 Kyushu Rosai Hospital Center for Preventive Medicine, Japan Occupational Health and Welfare Organization, Kitakyushu, Japan; 4 Department of Health Economics and Epidemiology Research, The University of Tokyo School of Public Health, Tokyo, Japan

**Keywords:** Stroke, Employment, Occupational factors, Rehabilitation, Speech disorders

## Abstract

**Objective:**

This study examined clinical, functional, and occupational factors associated with return to work within 18 months after stroke, specifically focusing on the impact of higher cortical dysfunction on return to work in the chronic phase.

**Methods:**

This prospective cohort study in 21 hospitals specializing in clinical and occupational health recruited consecutive working-age inpatients receiving acute care for their first stroke (*n* = 351). A unified database was used to extract patient information from hospital records at the time of admission, discharge, and follow-up at 18 months post-stroke. Cox proportional hazard regression analysis was conducted to determine clinical, functional, and occupational factors influencing return to work within 18 months.

**Results:**

Of 351 registered stroke patients (280 males, 71 females, mean age ± SD, 55.3 ± 7.2 years) who met inclusion criteria, 250 responded to the follow-up survey and 101 were lost to follow-up. Half (51 %) succeeded in returning to work during the 18-month follow-up after stroke onset. After adjusting for age, gender, and Barthel index at initial rehabilitation, the following factors were identified as significant predictors of a return to work: white-collar versus blue-collar occupation (hazard ratio (HR) 1.5; 95 % confidence interval (CI) 1.1–2.2), no aphasia (HR 3.0; 95 % CI 1.5–5.9), no attention dysfunction (HR 2.0; 95 % CI 1.0–4.0), and walking ability (HR 3.1; 95 % CI 1.3–7.1).

**Conclusions:**

This study indicated the importance of tailored rehabilitation to alleviate the impact of higher cortical dysfunction and to support return to work by stroke survivors.

## Introduction

A return to work plays an important role in the occupational health and rehabilitation of working-age post-stroke patients. Previous studies, including our own, identified determinants of early return to work in terms of functional and socioeconomic conditions of the patients (Saeki and Toyonaga 2010; Tanaka et al. 2011). These previous studies focused on the patient’s condition in the pre-stroke, hospitalized, and at-discharge periods, since these will predict the functional recovery which is expected within 3–6 months after onset (Bonita and Beaglehole 1988). However, the impact of higher cortical dysfunction has been poorly studied apart from a study by Tanaka et al. (2011) in which the authors identified that higher cortical dysfunction significantly reduced the chance of very early return to work within 1 month after discharge in those with mild physical impairment. Since the recovery in higher cortical function is likely to be observed several months after a stroke and into the chronic period after 6 months (Ferro and Crespo 1988), the influence of higher cortical dysfunction on return to work in the chronic phase could be more important than in the earlier phase. Furthermore, the earlier study did not specify what type of higher cortical function is related to return to work among those with different levels of physical impairment. In this study, we specifically focused on the impact of higher cortical dysfunction on return to work in the chronic phase, in addition to the functional and social factors discussed in previous studies. Since the rehabilitation of higher cortical dysfunction often requires a distinct set of resources compared with that required for physical dysfunction, we believe that the results of this study will provide information on the need for cognitive rehabilitation in the chronic stage of stroke recovery to enable return to work.

## Methods

### Participants

The study was performed on the same prospective cohort as in Tanaka et al. (2011). We included consecutive stroke patients aged between 15 and 64 years admitted to 21 hospitals between February 1, 2005 and July 31, 2006 for acute care of a first stroke event and who were in work at the time of the onset of stroke (*n* = 351). We excluded patients who were classified as a housewife or student. The hospitals belong to a nonprofit organization that provides special attention for occupation-related conditions and were founded by the Ministry of Health, Labour and Welfare of Japan. Stroke was diagnosed using the international classification of diseases, 10th revision (ICD-10) codes for cerebral hemorrhage, cerebral infarction, or subarachnoid hemorrhage.

### Outcome measure

The outcome was return to work after stroke, which was defined as active employment in formal paid work on a full-time or part-time basis which was identified at follow-up 18 months after the onset of stroke. The information was reported directly by patients, by physiatrists at the outpatient clinic interviewing patients, or by trained clerical staff interviewing patients by telephone at 18 months after onset.

### Procedures

A unified electronic data format was used to extract patient information from hospital records at the time of admission, discharge, and follow-up 18 months post-stroke. Data were collected on history and lifestyle factors, demographic factors, diagnostic factors, functional factors, and occupational factors. Physiatrists interviewed patients to obtain information regarding history and lifestyle factors at initial rehabilitation and collected clinical and diagnostic factors at discharge from medical records. Higher cortical dysfunction (brain impairment related to behavior, cognition, and language that cannot be explained by motor paralysis or sensory or perception disorders) was diagnosed by neurologists using the neurological examination based on higher cortical dysfunction diagnosis guidelines (Japanese Ministry of Health, Labour and Welfare 2007), the Standard Language Test of Aphasia (Japan Society for Higher Brain Dysfunction 2003), the Mini-Mental State Examination (Folstein et al. 1975), the line bisection test, and the Kohs block test. Radiologists independently and in a blinded manner made diagnoses regarding etiology, anatomical location, and size of stroke by neuroradiological imaging.

Occupational therapists evaluated functional factors with the modified Rankin scale (mRS) (van Swieten et al. 1988) and the Barthel index (BI) (Malloney and Barthel 1965). The BI is a measure of functional ability in personal care including self-care, bowel and bladder sphincter control, and mobility. Job type was classified according to the Japanese standard classification of occupations (Japanese Ministry of Health, Labour and Welfare 1997). We classified the following jobs as white collar: clerks, technicians, highly skilled professionals, directors, and managers. Unskilled workers, production-line/machine workers, drivers, skilled manual workers, farm/horticulture workers, and service workers were classified as blue collar.

Potential predictors of return to work within the 18-month follow-up after onset were selected according to the literature (Tanaka et al. 2011; Treger et al. 2007; Wozniak and Kittner 2002) and included age, gender, education, dysphagia, spasticity, visuospatial neglect (failing to report, respond, or orient to visual stimuli presented at the side opposite a brain lesion), aphasia (an acquired disorder of all language modalities, including verbal expression, auditory comprehension, written expression, and reading comprehension), attention dysfunction, memory dysfunction, intelligence dysfunction, etiological diagnosis, side of hemiplegia, BI at first rehabilitation, upper extremity function, walking ability, job type, work position, and mental stress at work.

This study was approved by the ethics committees of the Japan Occupational Health and Welfare Organization and the internal review board of each participating hospital. Written informed consent was obtained from each patient.

### Statistical analyses

Cox proportional hazard regression analysis was conducted with adjustment for three strong predictors of return to work, namely age, gender, and BI at initial rehabilitation, in order to select candidate variables from clinical, functional, and occupational factors for multivariable analysis. In a previous study, we used mRS at discharge because of a ceiling effect of BI in patients with relatively mild disability. In this study, we used BI at initial rehabilitation as an adjusting factor because it should more sensitively reflect the initial condition before rehabilitation. At this stage, *p* < 0.10 was used as the inclusion criterion. The Kaplan–Meier method was used to confirm the proportional hazard assumption of each variable. The selected candidate variables were further tested using forward stepwise regression analysis to obtain a final model to predict the likelihood of return to work within 18-month follow-up after stroke. In this final model, *p* < 0.05 was conventionally chosen as the level of statistical significance. Hazards ratios (HRs) were computed based on the estimated coefficients in Cox proportional hazard regression analysis. Since our previous study suggested that the impact of higher cortical dysfunction might depend on other conditions of the patient, we additionally tested whether the impact of higher cortical dysfunction was observed across job types, age strata, and initial severity of physical dysfunction. All statistical analyses were conducted using SPSS for Windows, version 19 (SPSS Inc., Chicago, IL, USA).

## Results

**Fig. 1:**
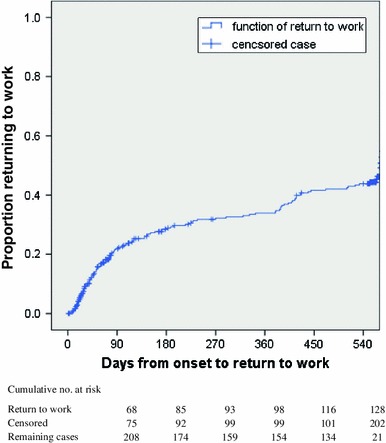
Proportion of patients returning to work during the 18 months after stroke onset

Of 351 registered stroke patients (280 males, 71 females, mean age ± standard deviation (SD), 55.3 ± 7.2 years, age range 21–64 years), met the inclusion criteria. As for etiology, 36 % were diagnosed with cerebral hemorrhage, 54 % with cerebral infarction, and 10 % with subarachnoid hemorrhage. At the 18-month follow-up, 250 responded to the survey (Table 1), while 101 were lost to follow-up. These two groups of responders and non-responders were not significantly different in terms of age (mean age ± SD: 55.4 ± 7.0 and 54.8 ± 7.6 years, respectively (*p* = 0.481)), BI at onset (mean score ± SD: 38.6 ± 37.6 and 42.8 ± 40.0, respectively (*p* = 0.382)), BI at initial rehabilitation (mean score ± SD: 55.3 ± 36.7 and 54.2 ± 39.0, respectively (*p* = 0.813)), and BI at discharge (mean score ± SD: 89.4 ± 21.7 and 90.1 ± 20.1, respectively (*p* = 0.774)). Among those who were followed-up, 128 patients (51 %: 51.5 % of men, 50 % of women) reported a successful return to work within 574 days after stroke onset (Fig. 1).

**Table 1:** Basic characteristics of subjects studied (*n* = 250)

Variables	Number of patients	Returned to work (%)	*p*
Demographic factors			
Gender			0.874
Male	202	51.5	
Female	48	50	
Education			0.03
College	44	70.5	
Junior college	18	55.6	
High school	123	49.6	
Less than high school	34	38.2	
Diagnostic factors			
Diagnosis			0.017
Cerebral hemorrhage	90	38.9	
Cerebral Infarction	133	57.1	
Subarachnoid hemorrhage	23	60.9	
Side of hemiplegia			0.007
Right	124	42.7	
Left	85	56.5	
Bilateral	7	28.6	
None	28	75	
Weakness in hemiplegic upper extremity			<0.001
Normal or mild	162	60.5	
Moderate	45	40	
Severe	39	23.1	
Weakness in hemiplegic lower extremity			<0.001
Normal or mild	188	60.1	
Moderate	45	26.7	
Severe	12	0	
Dysphasia			<0.001
No	227	54.6	
Yes	18	11.1	
Dysarthria			0.035
No	189	55	
Yes	57	38.6	
Aphasia			<0.001
No	201	57.7	
Yes	44	22.7	
Visuospatial neglect			<0.001
No	216	56	
Yes	29	13.8	
Apraxia			<0.001
No	228	54.4	
Yes	17	5.9	
Shoulder-hand syndrome			<0.001
No	229	54.6	
Yes	17	5.9	
Shoulder subluxation			<0.001
No	218	56.4	
Yes	28	10.7	
Spasticity			0.003
No	217	54.8	
Yes	29	24.1	
Depression			0.808
No	228	50.9	
Yes	18	55.6	
Attention dysfunction			<0.001
No	197	58.4	
Yes	48	22.9	
Memory dysfunction			<0.001
No	201	57.7	
Yes	43	20.9	
Intelligence dysfunction			0.001
No	209	56	
Yes	35	25.7	
Fatigability			0.002
No	182	57.1	
Yes	63	34.9	
Functional factors			
mRS* at initial rehabilitation			<0.001
0	3	33.3	
1	26	69.2	
2	42	71.4	
3	36	58.3	
4	71	49.3	
5	68	29.4	
mRS* at discharge			<0.001
0	24	62.5	
1	99	71.7	
2	63	49.2	
3	32	18.8	
4	22	13.6	
5	5	0	
Walking ability			<0.001
Independent	190	61.6	
Assisted	55	14.5	
Treatment factor			
Surgical operation			0.331
Yes	47	44.7	
No	195	53.3	
Occupational factors			
Job type			0.013
Blue collar	156	44.9	
White collar	94	67.1	
Work position			0.001
Manager	36	47.2	
Head of department	44	72.7	
Regular employee	115	52.2	
Other	41	29.3	
Full time or part time			0.127
Full time	198	55.1	
Part time	35	37.1	
Mental stress at work			0.011
No	167	45.5	
Yes	83	62.7	
Approach from physician to patient and family			0.019
Yes	106	60.4	
No	130	44.6	
Approach from physician to rehabilitation stuff			0.001
Yes	105	63.8	
No	132	43.2	
Employment status at discharge			<0.001
Employed	185	60	
Unemployed	41	12.2	
Patient wish for return to work			<0.001
Want	170	61.2	
Do not want	35	22.9	
Family wish for patient return to work			0.199
Want	131	58.8	
Do not want	17	41.2	
Satisfaction with social participation			<0.001
Yes	82	59.9	
No	55	55	
Collaboration with industrial physicians			0.062
Yes	23	78.3	
No	108	56.5	
Cooperation of workplace supervisors			0.016
Yes	50	78	
No	61	55.7	
Coordination of the work environment			1
Yes	10	70	
No	94	71.3	
Cooperation with vocational rehabilitation			0.41
Yes	17	76.5	
No	97	62.9	
Support of medical institutions on return to work			0.001
Yes	43	74.4	
No	131	45.8	

Total number of patients does not always equal 250 because of missing data

*Score 0* no symptoms, *Score 1* no significant disability despite symptoms, *Score 2* slight disability, *Score 3* moderate disability, *Score 4* moderately severe disability, and *Score 5* severe disability

* mRS—Rankin scale

After adjustment for age, gender, and BI at initial rehabilitation, the following variables showed significant associations with the return to work at 18-month follow-up: job type, work position, etiological diagnosis, upper extremity function, walking ability, spasticity, visuospatial neglect, aphasia, attention dysfunction, memory dysfunction, and intelligence dysfunction. Since etiological diagnosis and work position violated proportional hazard assumption in visual diagnosis with Kaplan–Meier curves, we excluded these variables in further analysis, leaving nine variables for further multivariable analysis (Table 2).

**Table 2:** Selected candidate variables associated with return to work within 18 months of onset after adjusting for age, gender, and Barthel index at initial rehabilitation

Variables	Reference	Hazard ratio	95 % confidence interval
Job type	White collar versus blue collar	1.6	1.1–2.2
Upper extremity function	Normal or mild versus severe	3.6	1.8–7.4
	Moderate versus severe	2.5	1.1–5.6
Walking ability	Independent versus dependent	4.8	2.2–10.6
Spasticity	No versus yes	2.9	1.3–6.3
Visuospatial neglect	No versus yes	4.7	1.7–12.9
Aphasia	No versus yes	3.3	1.7–6.3
Attention dysfunction	No versus yes	3.1	1.6–6.0
Memory dysfunction	No versus yes	2.8	1.4–5.6
Intelligence dysfunction	No versus yes	2.2	1.1–4.4

In stepwise Cox proportional hazard regression analysis, with adjustment for age, gender, and BI at initial rehabilitation, significant predictors of return to work at 18-month follow-up after stroke were job type, aphasia, attention dysfunction, and walking ability (Table 3). Specifically, those who had independent walking ability, were engaged in white-collar jobs, and were without aphasia and attention dysfunction were significantly more likely to return to work. Age (HR 0.96; 95 % confidence interval (CI) 0.94–0.98) and BI at initial rehabilitation (HR 1.01; 95 % CI 1.00–1.01) remained significant predictors after adjustment for walking ability, white-collar job, aphasia, and attention dysfunction.

**Table 3:** Multivariable model to predict return to work within 18 months after onset, analyzed by stepwise Cox proportional hazard analysis

Variables	Reference	Hazard ratio	95 % confidence interval
Job type	White collar versus blue collar	1.5	1.1–2.2
Aphasia	No versus yes	3.0	1.5–5.9
Attention dysfunction	No versus yes	2.0	1.0–4.0
Walking ability	Independent versus dependent	3.1	1.4–7.1

Adjusted for age, gender, and Barthel index at initial rehabilitation

In total, 311 cases were used in the analysis because of missing observations

Since job type, age, and BI at initial rehabilitation were significant influential factors, we further tested whether the impact of aphasia and attention dysfunction differed according to the levels of these properties. Stratified analysis by job type found that age, BI at initial rehabilitation, and no aphasia were significant predictors of return to work in white-collar workers, while age, BI at initial rehabilitation, walking capability, and no aphasia were significant among blue-collar workers. Lack of aphasia showed a HR for return to work of 4.0 (95 % CI 1.6–10.1) among white-collar workers and 2.8 (95 % CI 1.1–7.2) among blue-collar workers. The HR of no attention dysfunction did not differ by job type and was similar for white-collar and blue-collar workers. Stratification by age revealed that those aged 56 and younger had no aphasia, no attention dysfunction, and walking ability as significant predictors of return to work, while those aged 57 and over had age and BI at initial rehabilitation as significant predictors. The estimated HRs for return to work among younger age patients were 3.2 (95 % CI 1.5–6.7) for no aphasia and 2.8 (95 % CI 1.1–7.3) for no attention dysfunction. Finally, the stratification by BI scores at initial rehabilitation showed that age, no attention dysfunction, and walking ability were significant predictors among those with initial BI score less than 60, and age, gender, and no aphasia were significant predictors among those with initial BI score of 60 and greater. The HR of no aphasia was 3.2 (95 % CI 1.3–8.0) among those with milder physical dysfunction at initial status, while the HR of no attention dysfunction was 3.3 (95 % CI 1.3–8.1) among those with severe physical dysfunction.

## Discussion

In our previous study, it was identified that dysfunctions in attention, memory, and intelligence had a significant impact on very early return to work among those with only very mild physical impairment (Tanaka et al. 2011). In the current study, we additionally revealed that aphasia and attention dysfunction also had a significant impact on return to work within 18 months after stroke onset.

Previous studies indicated that neuropsychological impairments impact on vocational prognosis in the chronic stage of stroke. Kotila et al. (1984) showed that impairments in intelligence and memory had a major negative influence on return to work in the 12 months from stroke onset. Although there is little research on the relationship between attention dysfunction and return to work in stroke patients, some studies in traumatic brain injury cases reported that recovery of attention significantly improved return to work (Dawson et al. 2004; Mateer and Sira 2006). Vilkki et al. (2004) examined patients who had secondary cerebral infarction after aneurysmal subarachnoid hemorrhage and found that left-hemisphere infarctions causing deficits in verbal memory were likely to result in a failure to return to work within 1 year of the accident. Doucet et al. (2012) also reported that negative prognostic factors for a return to work after 3-year follow-up were language disorders (aphasia and dysarthria). The results of our study clearly indicated that patients without these factors had a significantly better chance of a return to work in the chronic phase.

The current study also suggested that the effect of aphasia and attention dysfunction varied according to concurrent conditions of stroke patients. Patients without aphasia showed a significantly higher chance of returning to work regardless of job types, suggesting that verbal communication with worksite colleagues could influence vocational prognosis in general (Black-Schaffer and Osberg 1990). In contrast, lack of attention dysfunction and aphasia was a significant factor among younger workers, but not among older workers. This difference according to age may indicate that differences in the levels of job complexity and demand may affect the chance of returning to work, especially among younger stroke survivors. It was also noteworthy that the role of attention dysfunction was significant among those with moderate to severe disability, while the role of aphasia was significant among the mildly disabled. Again, this may be explained by different job demands for patients with mild disability and for those with more severe disabilities. Demanding jobs with more complex communication requirements may be more likely to be assigned to patients with mild disability, while severely disabled patients may be assigned less demanding jobs that may not require so much communication and attention capabilities. Although the explanation above is only speculative because we did not have detailed information on the nature of the patients’ jobs, our findings may indicate the need of tailored job reallocation and rehabilitation programs according to patient’s age, former job, and remaining functions after stroke.

Persons with more skilled forms of employment may have a greater chance of returning to work because such forms of employment may allow an appropriate redesign of working conditions even for patients in the chronic stage of stroke recovery. The identified impact of aphasia and attention dysfunction may be alleviated through intensive rehabilitation treatment of higher functions. Hinckley (2002) noted that 62 % of chronic aphasia patients from an intensive treatment program were in employment 2 years after discharge. Aphasia rehabilitation may also promote community reintegration, workplace flexibility, and enhancement of social support to the patients that further enables the person with aphasia to return to a former job.

The current study confirmed that job type remained significantly related to the chance of employment after 18 months from onset as well as to very early return to work, which was consistent with findings in previous studies in Japan and in other countries (Saeki et al. 1993; Howard et al. 1985; Hannerz et al. 2011; Vestling et al. 2003). Some studies reported that age was not related to very early return to work, but our study found that younger age was significantly associated with a return to employment within 18 months. Previous rehabilitation studies suggested that there were no differences in the chance of recovery from walking disability, attention dysfunction, and aphasia according to age, and they recommended intensive rehabilitation regardless of patient age (Pickersgill and Lincoln 1983; Luk et al. 2006; Denti et al. 2008). However, several studies, including this study, revealed that older age was related to a lower probability of returning to work in the chronic stage (Howard et al. 1985; Hannerz et al. 2011; Saeki 2000, Busch et al. 2009; Wozniak et al. 1999). We speculate that social as well as physiological conditions may play a role in employment rehabilitation of older patients who face restrictive social conditions for labor participation. Investigation of social aspects of rehabilitation into the working environment is warranted to further facilitate return to work of stroke patients irrespective of age.

In our analysis, the BI and walking ability in the early phase were related to return to work within 18 months. In our previous study on early return to work (Tanaka et al. 2011), we used the mRS at discharge as a predictor of return to work. Since walking and functional abilities reflected in BI are influential factors determining the level of the mRS, the results confirmed that functional and walking disability similarly affected the chance of return to work in very early as well as in the chronic phase.

We could not use the factors of family wish for patient return to work, collaboration with industrial physicians, cooperation of workplace supervisors, coordination of the work environment, provision of vocational rehabilitation, and support of medical institutions on return to work as independent variables in the multivariate analysis because of the large number of missing observation. The impact of support from patient’s family and former work place on return to work deserves further investigation in future research.

This study had several limitations. First, at the 18-month follow-up, 250 responded to the survey, while 101 were lost to follow-up. Of the 101 patients, four had died and 21 survived, but did not respond, while the other 76 patients had lost contact. There was no significant difference between responders and lost patients in terms of age, BI at onset, BI at initial rehabilitation, and BI at discharge. However, the high attrition rate could lead to bias in our analysis.

Second, there was a considerable amount of missing information on non-medical factors that may affect the likelihood of return to work, such as family wish for patient return to work and collaboration with industrial physicians. Inclusion of non-medical support from family and workplace might have modified the final model in predicting success in return to work 18 months after stroke.

Third, although our results indicate rehabilitation program for higher cortical dysfunction may be effective to enhance the chance of return to work among young patients with mild physical disability, we could not directly show cost-effectiveness of such program due to our data limitation, which remains to be articulated in future research.

In conclusion, specific types of higher cortical dysfunction such as aphasia and attention dysfunction as well as walking ability and job type had a significant impact on return to work among stroke survivors within 18 months of onset, after adjustment for age, gender, and physical dysfunction at initial rehabilitation. The impact of higher cortical dysfunction was more likely to be observed among young and mildly disabled patients, suggesting the need for a tailored rehabilitation program and job redesign for patients with higher cortical dysfunction after stroke. This study indicated the importance of cognitive rehabilitation to alleviate the impact of higher cortical dysfunction and to support return to work by stroke survivors.
